# Differential age-related patterns in the associations of LDL-C, TG/HDL-C ratio, and carotid plaque: a large-scale cross-sectional study in a Chinese health check-up population

**DOI:** 10.3389/fcvm.2026.1887660

**Published:** 2026-07-20

**Authors:** Zeda Li, Biqiong Zhu, Kui Chen, Xiangdang Long, Kang Zhao

**Affiliations:** 1 Hunan Provincial People's Hospital and The First-Affiliated Hospital of Hunan Normal University, Changsha, China; 2 The Third Xiangya Hospital, Central South University, Changsha, China

**Keywords:** age stratification, carotid plaque, interaction, LDL-C, TG/HDL-C

## Abstract

**Background:**

While the associations of low-density lipoprotein cholesterol (LDL-C) and the triglyceride-to-high-density lipoprotein cholesterol ratio (TG/HDL-C) with carotid atherosclerosis are known, their age-varying trajectories remain unclear. This study aimed to characterize the age-specific heterogeneity in these associations using a large Chinese health check-up cohort.

**Methods:**

We retrospectively enrolled 23,058 adults undergoing carotid ultrasonography. After exclusions, 21,227 participants were included. Multivariate logistic regression evaluated the independent associations of LDL-C and TG/HDL-C with carotid plaque. Age-stratified analyses (<40, 40–49, 50–59, 60–69, ≥70 years) and interaction tests were performed. Sensitivity analyses included substituting non-HDL-C for LDL-C and the triglyceride-glucose index (TyG index) for TG/HDL-C. Associations with hypoechoic plaques were also assessed.

**Results:**

The carotid plaque detection rate was 31.4% (6,657/21,227). Both LDL-C (OR = 1.12, 95% CI: 1.08–1.17, *P* < 0.001) and TG/HDL-C (OR = 1.11, 95% CI: 1.07–1.15, *P* < 0.001) were independently associated with plaque. However, their age patterns diverged significantly. The association of LDL-C was strongest in participants aged <40 years (OR = 1.51, 95% CI: 1.30–1.76) but attenuated with age, becoming non-significant in those ≥60 years (*P* for interaction <0.001). Conversely, the TG/HDL-C association remained stable across age groups, with the numerically highest ORs observed in the <40 and 50–59 age groups (OR = 1.20 and 1.15, respectively), though the age interaction was not significant (*P* = 0.544). Within the plaque population, LDL-C correlated with hypoechoic plaques (OR = 1.23, 95% CI: 1.16–1.30), while the TyG index did not.

**Conclusion:**

LDL-C and TG/HDL-C exhibit distinct age-modified patterns of association with carotid plaque. The LDL-C association is most pronounced in younger individuals, whereas the TG/HDL-C association remains relatively stable across age strata. These cross-sectional findings generate the hypothesis that age-specific lipid assessment strategies may warrant prospective evaluation.

## Background

Carotid atherosclerosis serves as a pivotal surrogate marker for systemic atherosclerosis and represents a robust predictor of ischemic stroke and coronary heart disease (1, 2). As a direct manifestation of focal atherosclerotic burden, carotid plaque is readily detectable in routine health check-ups and closely correlates with the future risk of cardio-cerebrovascular events (3, 4). Thus, elucidating independent and manageable predictors of carotid atherosclerosis carries significant clinical utility in mitigating the onset of cardiovascular events.

Low-density lipoprotein cholesterol (LDL-C) is widely regarded as a core pathogenic factor in atherosclerosis. Extensive evidence from clinical trials and Mendelian randomization studies strongly supports a causal link with adverse cardiovascular outcomes (5, 6). However, emerging evidence suggests that the atherogenic impact of LDL-C may be age-dependent. Longitudinal research underscores that the detrimental effect of elevated LDL-C on cardiovascular events is most critical among younger individuals, with this association weakening as age advances (7, 8). Whether this age-dependent phenomenon extends to carotid plaque—a critical pre-clinical phenotype—remains insufficiently characterized, particularly in the context of formal interaction testing within large-scale population data.

Beyond standard LDL-C metrics, the TG/HDL-C ratio captures the metabolic signature of dyslipidemic profiles that fuel atherosclerosis; it stands as an independent determinant of insulin resistance, small dense LDL burden, and excess cardiovascular risk not explained by traditional factors (9, 10). Crucially, the TG/HDL-C ratio can be derived directly from standard lipid profiles without incurring additional costs, rendering it a highly practical tool for resource-limited primary care settings. However, whether its association with carotid plaque is independent of LDL-C, and whether this association exhibits similar age heterogeneity, remains insufficiently defined (11, 12). Moreover, the TyG index—a non-invasive proxy synthesizing fasting glycemia and triglyceride levels (13–15)—was included as a secondary sensitivity marker to evaluate whether a composite measure incorporating glucose would yield associations comparable to the TG/HDL-C ratio, given that both reflect insulin resistance. Notably, most previous investigations have treated age merely as a confounder to be adjusted for, rather than as a potential effect modifier to be explored via stratification and interaction analyses (16, 17). Such an approach risks masking the true strength of lipid-related associations within specific age strata. Consequently, LDL-C and TG/HDL-C capture distinct atherogenic pathways: LDL-C directly reflects cholesterol burden in the arterial wall, whereas the TG/HDL-C ratio serves as an integrative marker of metabolic dysregulation, insulin resistance, and the predominance of small dense lipoprotein particles. Their differential age-dependent associations may provide insights into the changing determinants of atherosclerosis over the life course. Accordingly, this cross-sectional study was designed to explore whether the associations of these two lipid measures with carotid atherosclerosis differ across the age spectrum, with the aim of generating hypotheses for future prospective investigation. Specific objectives included: (1) evaluating and comparing their independent associations with carotid plaque; (2) delineating the age-dependent trajectories of these associations through stratified analyses and formal interaction tests; and (3) assessing their differential roles in plaque vulnerability by examining associations with hypoechoic plaques.

## Methods

### Participants

We conducted a retrospective cross-sectional analysis involving consecutive adult subjects who received standardized health screenings and carotid ultrasounds at the Health Promotion Center of a major tertiary-level hospital in the province. Of 23,058 individuals initially screened, 21,227 were ultimately included in the final analysis after applying the exclusion criteria: (1) age <18 years; (2) history of established cardio-cerebrovascular events (including myocardial infarction, stroke, or revascularization procedures) documented in medical records or self-reported; (3) missing data on key variables, including carotid ultrasound parameters, fasting lipid profiles, fasting glucose, blood pressure, height, or weight. A detailed overview of the participant disposition is provided in Supplementary Figure S1. The research protocol adhered to the ethical principles outlined in the Declaration of Helsinki and received ethics clearance from the Institutional Review Board of Hunan Provincial People's Hospital (Approval No. [2026]-131).Owing to the retrospective design and the utilization of de-identified data, the Institutional Review Board exempted this study from the obligation to obtain informed consent. The reporting of this cross-sectional study aligns with the Strengthening the Reporting of Observational Studies in Epidemiology (STROBE) guidelines (18, 19).

### Data source

All participants underwent standardized health examinations. Demographic information, height, and weight were collected, and body mass index (BMI) was calculated. Resting SBP and DBP were recorded. Following a minimum 12-hour fast, venous blood was sampled for automated biochemical analysis of serum TC, TG, HDL-C, and fasting glucose. LDL-C was subsequently derived via the Friedewald equation (LDL-C = TC-HDL-C-TG/2.2) for specimens with TG concentrations not exceeding 4.5 mmol/L (20, 21). Non-HDL-C was derived by subtracting HDL-C from TC. The TG/HDL-C ratio was determined by dividing TG by HDL-C. Furthermore, the TyG index was computed using the natural logarithm of the product of TG and fasting glucose (mg/dL) divided by two (22–24). Medication history was retrieved from the electronic health check-up system and verified via clinical inference. Suspected statin use was defined as meeting either of the following criteria: (1) self-reported use of lipid-lowering medication, or (2) LDL-C < 1.8 mmol/L, with other known etiologies for low LDL-C (e.g., cirrhosis, malignancy, severe malnutrition) excluded through review of medical history records in the check-up system and relevant biochemical parameters (e.g., liver function tests, albumin). In the primary analysis, all participants were retained to maximize statistical efficiency. To evaluate the influence of this decision, two pre-specified sensitivity analyses were conducted: (1) additionally adjusting for suspected statin use as a covariate in the full cohort; and (2) excluding all suspected statin users and repeating the primary analyses.

### Carotid ultrasound assessment and outcome definitions

Imaging of the carotid arteries was acquired by certified sonographers via a Philips EPIQ 7 platform, incorporating a high-frequency linear transducer (5–12 MHz). Adhering to established protocols, we performed a systematic evaluation of the bilateral common carotid arteries, bifurcations, and extracranial internal carotid arteries. IMT measurements of the distal common carotid artery were obtained according to standardized protocols, focusing on the far wall up to 1 cm below the carotid bifurcation. The maximum IMT (IMTmax) was defined as the greater value between the left and right sides. IMT thickening was defined as IMTmax ≥ 1.0 mm (25–27). In alignment with the Mannheim Consensus, a carotid plaque was identified if any of the following criteria were met: an intraluminal projection of ≥0.5 mm, a focal IMT ≥50% thicker than the surrounding vessel wall, or an absolute IMT value >1.5 mm (28). Hypoechoic plaques were defined as those with a hypoechoic area constituting ≥50% of the total plaque area (29). All images were assessed by a pair of expert sonographers who were kept unaware of patient information. Discordant findings were reconciled by mutual agreement. However, it should be noted that inter-observer agreement for hypoechoic plaque assessment was not formally quantified using kappa statistics, representing a limitation in the quality control of image interpretation. The primary outcome was the presence or absence of carotid plaque. The secondary outcome was the presence of hypoechoic plaques among the subpopulation with established carotid plaques.

### Statistical analysis

Given the distribution of the data, continuous measures were reported as medians with interquartile intervals, and categorical attributes were expressed as absolute frequencies and proportions. Non-parametric inferences for continuous variables were conducted using the Mann–Whitney *U*-test(two groups) or Kruskal–Wallis H test (>2 groups), alongside the Pearson chi-square test for categorical variables. Multivariable binary logistic regression was implemented to ascertain the independent correlates of carotid plaque. The primary model incorporated covariates including age, sex, BMI, systolic blood pressure, fasting glucose, LDL-C, and the TG/HDL-C ratio (all treated as continuous measures). Multicollinearity diagnostics were performed using the variance inflation factor (VIF), with values below 5.0 denoting acceptable tolerance. The discriminatory performance of the models was quantified by the area under the ROC curve (AUC).

To investigate age heterogeneity, participants were stratified into five age groups (<40, 40–49, 50–59, 60–69, and ≥70 years), and multivariable logistic regression models were fitted within each stratum, adjusting for continuous age and other covariates. Age modification was formally tested using multiplicative interaction terms. Specifically, product terms of LDL-C × age and TG/HDL-C ratio × age were entered into logistic regression models, with age modeled as a continuous variable centered at its mean. Likelihood ratio tests (LRT) were used to compare models with and without the interaction term, to evaluate the incremental contribution of the interaction. For the <40 age group, where the number of plaque events was limited (*n* = 284), Firth penalized likelihood regression was applied to reduce small-sample bias and ensure stable estimation; this method was also applied in sensitivity analyses when event counts were low.

The potential for non-linearity was assessed by fitting restricted cubic spline (RCS) models with four knots corresponding to the 5th, 35th, 65th, and 95th percentiles of the predictor distribution (LDL-C: 1.78, 2.76, 3.42, 4.60 mmol/L; TG/HDL-C ratio: 0.37, 0.813, 1.411, 3.261). The reference (OR = 1.0) was set at the median (LDL-C: 3.09 mmol/L; TG/HDL-C ratio: 1.077). Non-linearity was tested by likelihood ratio test comparing models with and without the spline terms.

To visually illustrate the age-trajectory differences, adjusted odds ratios (ORs) for carotid plaque per 1-unit increment in LDL-C (1 mmol/L) and per 1-unit increment in the TG/HDL-C ratio were estimated across continuous age using models incorporating interaction terms. The corresponding OR curves with 95% confidence bands were plotted on the same coordinate axis.

To systematically assess the potential impact of statin-related bias and unmeasured confounding, we performed the following pre-specified sensitivity analyses: (1) additionally adjusting for suspected statin use as a covariate in the full cohort; (2) excluding all suspected statin users and repeating the primary analyses; (3) substituting non-HDL-C for LDL-C; (4) excluding individuals with fasting glucose ≥7.0 mmol/L; (5) further adjusting for IMTmax in the main model; (6) substituting the TyG index for the TG/HDL-C ratio; and (7) calculating E-values for the primary positive associations to quantify the minimum strength of association that an unmeasured confounder would need to have with both the exposure and the outcome to fully explain away the observed association. E-values were computed using the method of VanderWeele and Ding (30). Additionally, among the subpopulation with established plaques, multivariable logistic regression was performed with hypoechoic plaque as the outcome.

Statistical inference was based on two-sided tests, with results deemed significant at *P* < 0.05. All statistical procedures were carried out utilizing the R programming language (v4.5.3).

## Results

### Baseline characteristics

**Figure 1:**
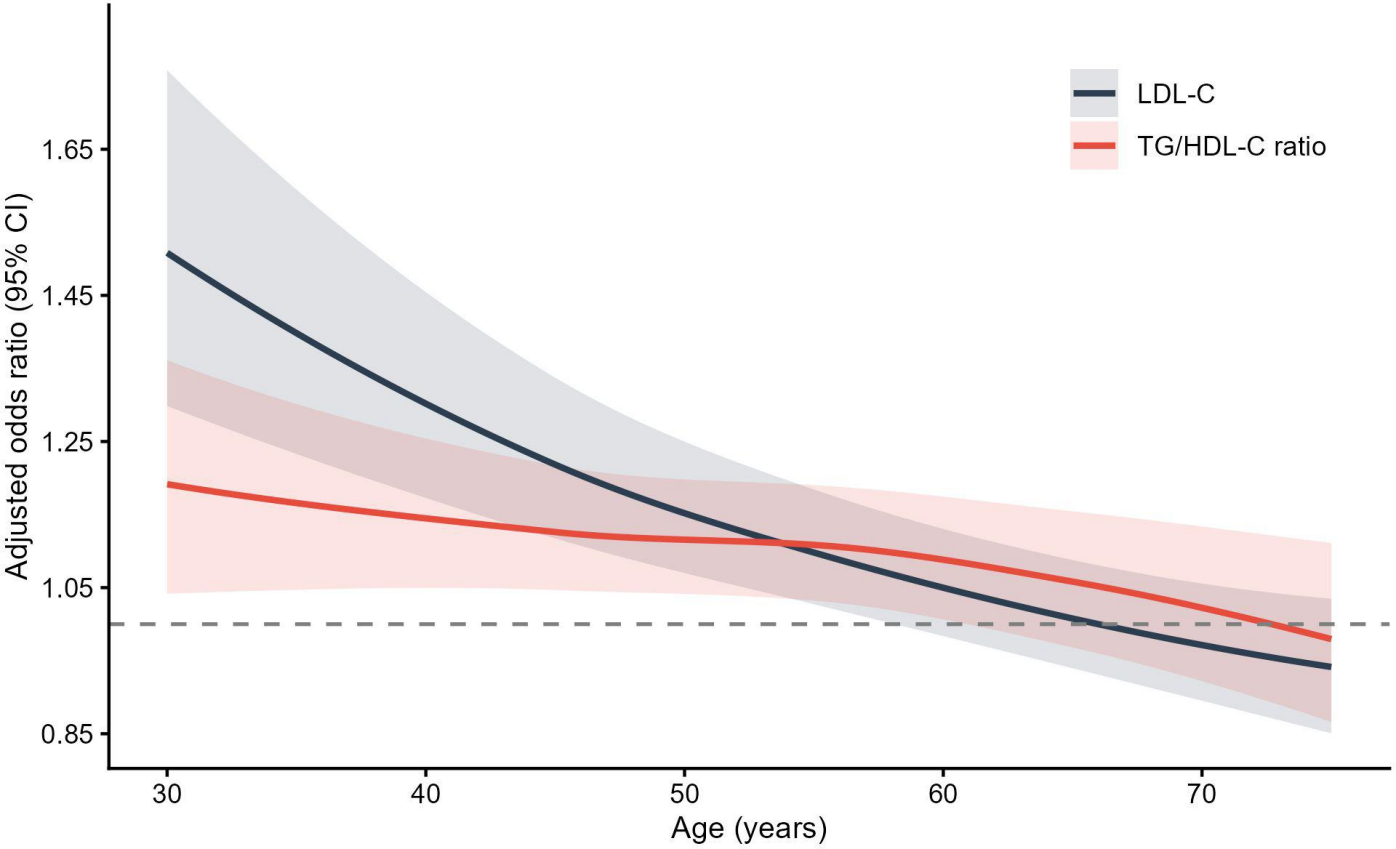
Prevalence of carotid plaque across age groups and sex.

The study cohort (*n* = 21,227) exhibited a median age of 52 years (IQR: 40–62), with a male predominance of 62.7%. Carotid plaque was identified in 6,657 individuals (prevalence: 31.4%). Table 1 outlines the baseline demographic and clinical profiles stratified by plaque status. Relative to participants free of plaque, those with carotid lesion were characterized by advanced age, a predominance of males, and elevated SBP, fasting glucose, LDL-C, and TG/HDL-C ratios (all *P* < 0.001). Furthermore, the prevalence of carotid plaque exhibited a marked ascending trend with advancing age, and consistently remained higher in males than in females across all age strata Figure 1. Prevalence of carotid plaque across age groups and sex.

**Table 1:** Baseline characteristics stratified by carotid plaque status.

Characteristic	No Plaque	Plaque	*p*-value^2^
	*N* = 14,570^1^	*N* = 6,657^1^	
Age (years)	45 [37, 55]	60 [52, 70]	<0.001
Gender			<0.001
Female	5,753 (39%)	2,154 (32%)	
Male	8,817 (61%)	4,503 (68%)	
Height (cm)	166 [159, 171]	165 [158, 170]	<0.001
Weight (kg)	66 [58, 75]	65 [58, 73]	<0.001
BMI (kg/m^2^)	24.2 [22.0, 26.3]	24.3 [22.3, 26.2]	0.038
SBP (mmHg)	123 [112, 134]	132 [121, 144]	<0.001
DBP (mmHg)	74 [67, 83]	77 [70, 85]	<0.001
Glucose (mmol/L)	5.20 [4.91, 5.59]	5.46 [5.08, 6.05]	<0.001
Total cholesterol (mmol/L)	5.01 [4.42, 5.64]	5.17 [4.47, 5.85]	<0.001
Triglycerides (mmol/L)	1.32 [0.92, 1.93]	1.40 [1.02, 1.99]	<0.001
HDL-C (mmol/L)	1.26 [1.08, 1.50]	1.25 [1.07, 1.46]	0.003
LDL-C (mmol/L)	3.06 [2.52, 3.63]	3.15 [2.52, 3.76]	<0.001
Non-HDL-C (mmol/L)	3.70 [3.12, 4.32]	3.86 [3.19, 4.53]	<0.001
TG/HDL-C ratio	1.05 [0.64, 1.71]	1.13 [0.72, 1.74]	<0.001
LDL-C/HDL-C ratio	2.44 [1.86, 3.04]	2.50 [1.89, 3.13]	<0.001
IMTmax (mm)	0.70 [0.70, 0.90]	1.00 [0.90, 1.10]	<0.001
Suspected statin use	268 (1.8%)	416 (6.2%)	<0.001

Continuous and categorical variables are expressed as median [IQR] and *n* (%), respectively. *P* values were obtained via Wilcoxon rank-sum or Pearson's chi-squared tests. BMI, DBP, HDL-C, IMTmax, LDL-C, SBP, TG. LDL-C (Friedewald equation) was excluded for TG > 4.5 mmol/L (*n* = 891), leaving 21,227 for analysis.

**Table 2:** Multivariable logistic regression for carotid plaque.

Characteristic	OR (95% CI)	*p*-value
Age (per 1 year)	1.09 (1.09–1.09)***	<0.001
Female	–	–
Male	1.68 (1.56–1.81)***	<0.001
BMI (per 1 kg/m^2^)	0.97 (0.96–0.99)***	<0.001
SBP (per 1 mmHg)	1.01 (1.00–1.01)***	<0.001
Glucose (per 1 mmol/L)	1.04 (1.01–1.07)**	0.002
LDL-C (per 1 mmol/L)	1.12 (1.08–1.17)***	<0.001
TG/HDL-C ratio (per 1 unit)	1.11 (1.07–1.15)***	<0.001

Odds ratios are adjusted for all other covariates in the model.

*p < 0.05; **p < 0.01; ***p < 0.001.

AUC, area under the receiver operating characteristic curve; BMI, body mass index; CI, confidence interval; LDL-C, low-density lipoprotein cholesterol; OR, odds ratio; SBP, systolic blood pressure; TG/HDL-C, triglyceride to high-density lipoprotein cholesterol ratio; VIF, variance inflation factor.

VIF values: Age = 1.24; Sex = 1.16; BMI = 1.29; SBP = 1.24; Glucose = 1.09; LDL-C = 1.01; TG/HDL-C = 1.22. C-statistic (AUC) = 0.805 (95% CI: 0.799–0.811). Interaction terms were tested with likelihood ratio tests comparing models with and without the lipid × age product term. Age was mean-centered and entered as a continuous variable. *P* for interaction (LDL-C × Age) < 0.001; *P* for interaction (TG/HDL-C × Age) = 0.544.

### Independent associations with carotid plaque

**Figure 2:**
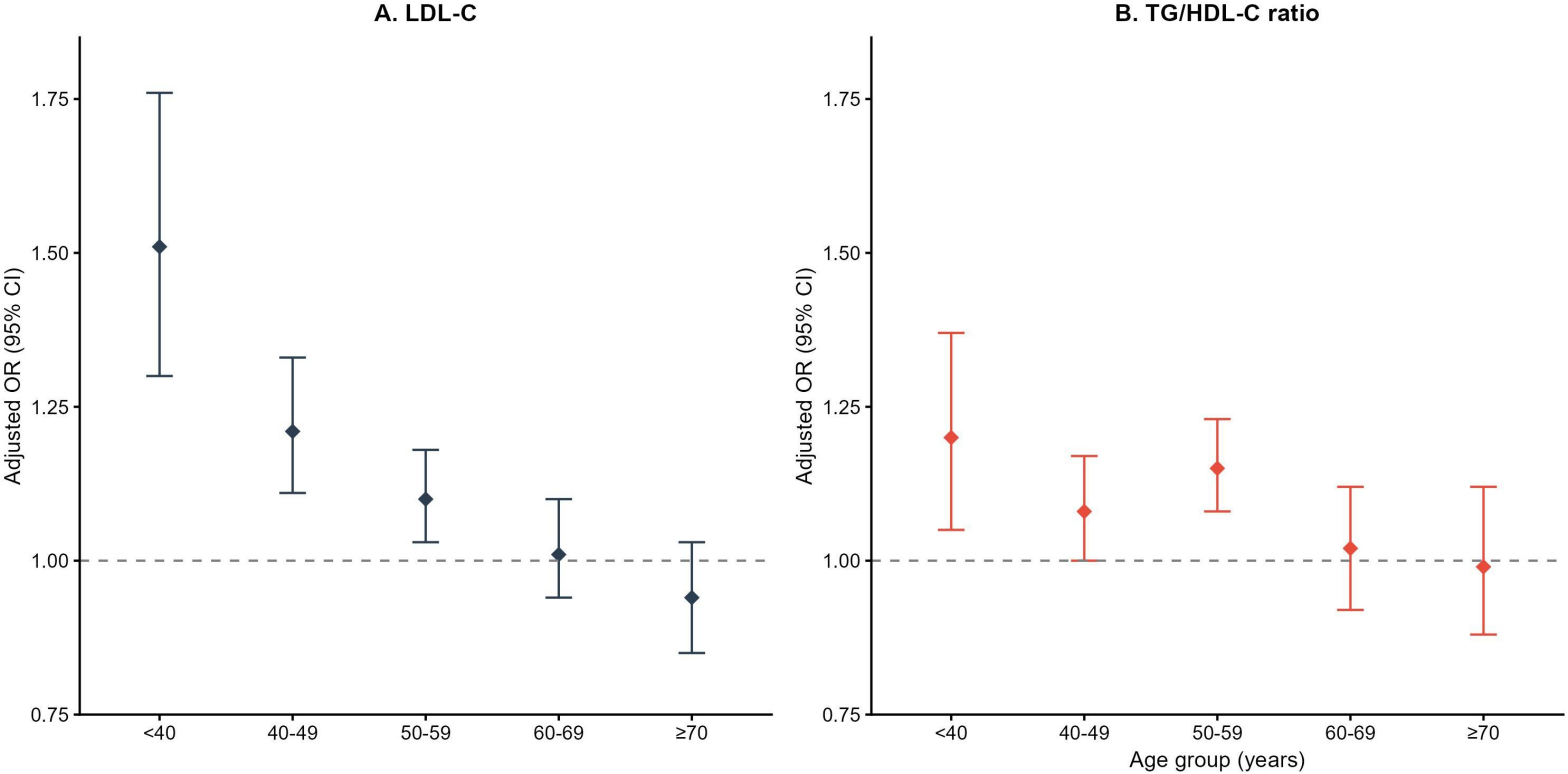
Multivariable-adjusted odds ratios (ORs) with corresponding 95% confidence intervals (CIs) for factors linked to carotid plaque are presented in Table 2. The reference line (OR = 1.0) is depicted by the dashed line. BMI, body mass index; CI, confidence interval; LDL-C, low-density lipoprotein cholesterol; OR, odds ratio; SBP, systolic blood pressure; TG/HDL-C, triglyceride to high-density lipoprotein cholesterol ratio.

Fully adjusted logistic regression models demonstrated that each 1 mmol/L increment in LDL-C conferred a 12% higher odds of prevalent plaque (OR = 1.12, 95% CI: 1.08–1.17; *P* < 0.001) (Table 2, Figure 2. Analogously, each 1-unit rise in the TG/HDL-C ratio was independently linked to an 11% elevation in plaque odds (OR = 1.11, 95% CI: 1.07–1.15; *P* < 0.001). The discrimination of the fully adjusted model yielded a C-statistic of 0.805 (95% CI: 0.799–0.811). Formal multiplicative interaction tests showed that LDL-C × age was significant (*β* = −0.0069, SE = 0.0016, 95% CI: −0.0100 to −0.0038, *P* < 0.001; LRT *χ*^2^ = 15.6, *P* < 0.001), supporting an age-attenuated association. TG/HDL-C × age was not significant (*β* = −0.0010, SE = 0.0016, 95% CI: −0.0041 to 0.0021, *P* = 0.544; LRT *χ*^2^ = 0.37, *P* = 0.544), indicating a stable association across age groups.

### Distinct age-modified patterns of lipid parameters

**Figure 3:**
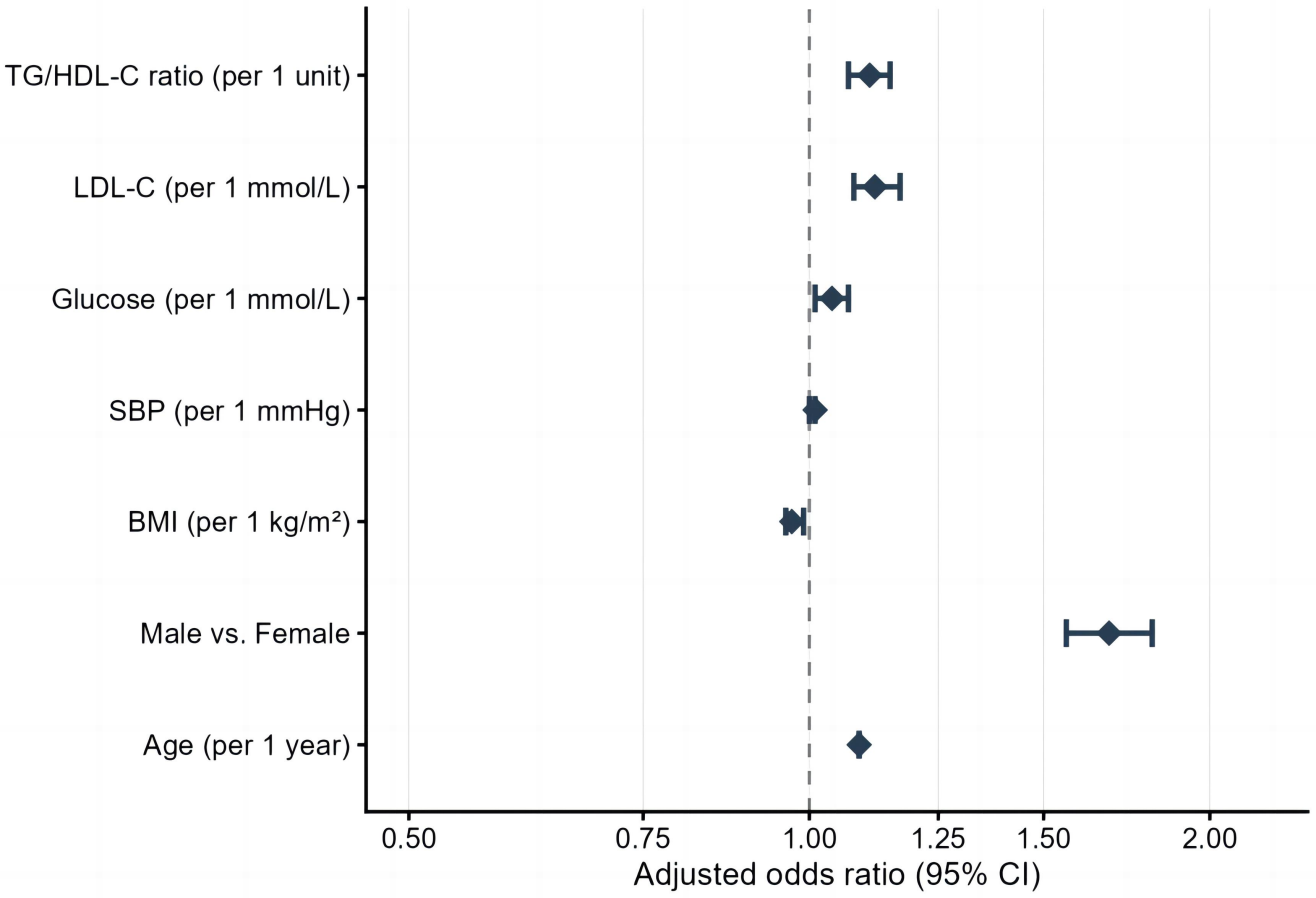
Age-specific associations of LDL-C **(A)** and the TG/HDL-C ratio **(B)** with carotid plaque. Covariates included continuous age, sex, BMI, SBP, and fasting glucose. BMI, body mass index; CI, confidence interval; LDL-C, low-density lipoprotein cholesterol; OR, odds ratio; SBP, systolic blood pressure; TG/HDL-C, triglyceride to high-density lipoprotein cholesterol ratio.

Age-stratified analyses clearly delineated the divergent associations of these lipid parameters with carotid plaque (Table 3, Figure 3).The magnitude of association for LDL-C demonstrated a clear attenuation with advancing age. The adjusted OR was highest in participants aged <40 years (OR = 1.51, 95% CI: 1.30–1.76, *P* < 0.001), decreased to 1.21 (95% CI: 1.11–1.33) in the 40–49 year-old group, and further declined to 1.10 (95% CI: 1.03–1.18) in the 50–59 year-old group. Notably, the association became statistically non-significant in the older age strata, with ORs of 1.01 (95% CI: 0.94–1.10) for those aged 60–69 years and 0.94 (95% CI: 0.85–1.03) for those aged ≥70 years. Firth penalized likelihood regression was applied to the <40 age group to ensure stable estimation given the limited number of events (*n* = 284). In stark contrast, the association between the TG/HDL-C ratio and carotid plaque remained relatively stable across age groups. with the numerically highest ORs observed in the <40 and 50–59 age groups (OR = 1.20 and 1.15, respectively).

**Table 3:** Age-stratified multivariable associations of LDL-C and TG/HDL-C ratio with carotid plaque.

Age Group (years)	*N*	Plaque Events	Prevalence (%)	LDL-C OR (95% CI)	TG/HDL-C OR (95% CI)
<40	5,324	284	5.3	1.51 (1.3–1.76)***	1.2 (1.05–1.37)**
40–49	5,033	935	18.6	1.21 (1.11–1.33)***	1.08 (1–1.17)
50–59	5,317	1,980	37.2	1.1 (1.03–1.18)**	1.15 (1.08–1.23)***
60–69	3,151	1,782	56.6	1.01 (0.94–1.1)	1.02 (0.92–1.12)
≥70	2,402	1,676	69.8	0.94 (0.85–1.03)	0.99 (0.88–1.12)

Values are adjusted odds ratios with 95% confidence intervals. Models within each age stratum were adjusted for continuous age, sex, BMI, SBP, and fasting plasma glucose. Detailed baseline characteristics by age stratum are provided in Supplementary Table S10. Prevalence was calculated as the proportion of participants with carotid plaque within each age stratum.

BMI, body mass index; CI, confidence interval; LDL-C, low-density lipoprotein cholesterol; OR, odds ratio; SBP, systolic blood pressure; TG/HDL-C, triglyceride to high-density lipoprotein cholesterol ratio.

*p < 0.05; **p < 0.01; ***p < 0.001. Firth penalized likelihood regression was applied to the <40 age group to ensure stable estimation given the limited number of events.

**Figure 4:**
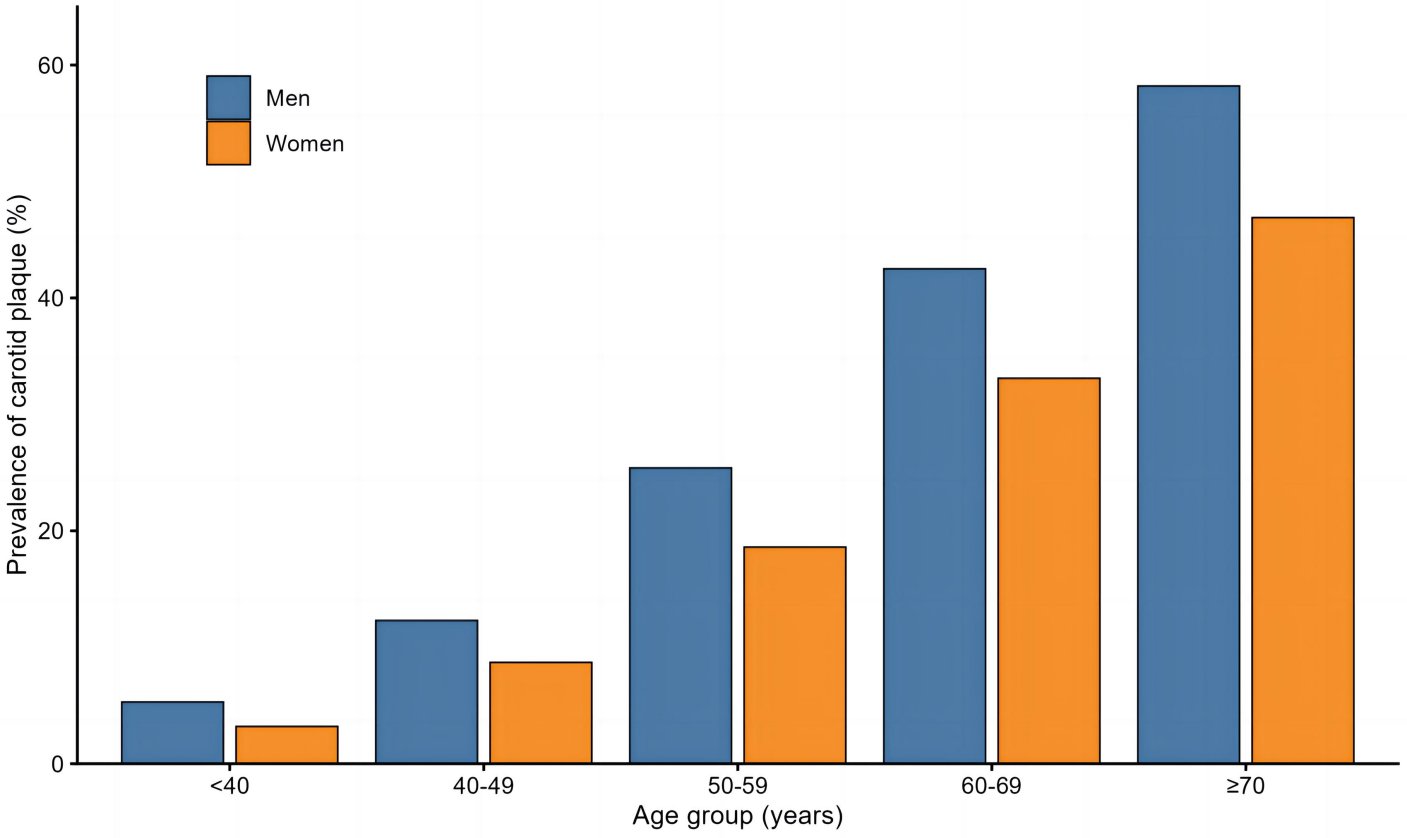
Age-varying adjusted ORs for carotid plaque per 1 mmol/L LDL-C and per 1-unit TG/HDL-C ratio. Shaded bands denote 95% CIs. BMI, body mass index; CI, confidence interval; LDL-C, low-density lipoprotein cholesterol; OR, odds ratio; SBP, systolic blood pressure; TG/HDL-C, triglyceride to high-density lipoprotein cholesterol ratio.

The continuous age-trajectory curves (Figure 4) visually encapsulated these disparities. The adjusted OR for LDL-C demonstrated a monotonic decline with advancing age. In contrast, the OR trajectory for the TG/HDL-C ratio remained relatively flat, exhibiting a modest bulge during the middle-age segment.

### Dose-response patterns

**Figure 5:**
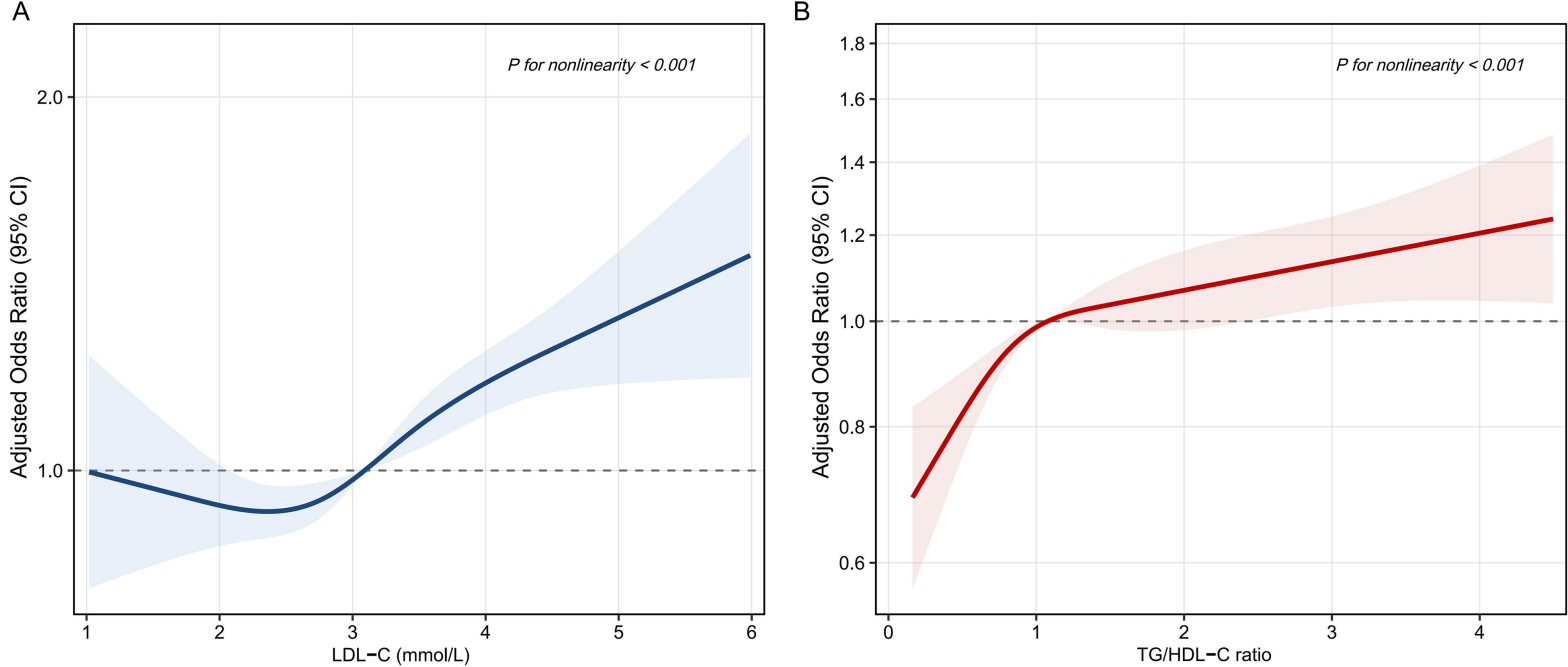
Visualization of non-linear exposure-response associations between carotid plaque and LDL-C **(A)** and the TG/HDL-C ratio **(B)** using restricted cubic splines. Adjusted ORs are depicted by solid lines, with shaded bands indicating 95% CIs; marginal tick marks display the data distribution. Both associations exhibited significant non-linearity (*P* for nonlinearity <0.001). RCS models were fitted with 4 knots at the 5th, 35th, 65th, and 95th percentiles. The reference (OR = 1.0) was set at the median (LDL-C: 3.09 mmol/L; TG/HDL-C ratio: 1.077). Shaded areas represent 95% CIs, which widen at extreme values. Non-linearity was assessed by likelihood ratio test. The apparent inflection points (approximately 2.5–3.0 mmol/L for LDL-C and 0.8–1.0 for TG/HDL-C ratio) are descriptive features of the fitted spline model and should not be interpreted as discrete clinical thresholds.

Restricted cubic spline (RCS) modeling revealed distinct, non-linear positive exposure-response gradients for both LDL-C and the TG/HDL-C ratio in relation to carotid plaque (Figure 5). The tests for non-linearity yielded highly significant *P*-values for both LDL-C (*P* < 0.001) and the TG/HDL-C ratio (*P* < 0.001). The RCS curves showed that the risk for carotid plaque began to rise notably at approximately 2.5–3.0 mmol/L for LDL-C and at approximately 0.8–1.0 for the TG/HDL-C ratio, with the slopes flattening beyond the 65th percentile (LDL-C: 3.42 mmol/L; TG/HDL-C ratio: 1.411). At extreme high values, the 95% confidence intervals widened considerably, indicating greater uncertainty in this range.

### Sensitivity analyses and subgroup results

To evaluate potential bias introduced by suspected statin use, we implemented three complementary analytical strategies. When additionally adjusting for suspected statin use in the full cohort, the LDL-C–plaque association exhibited a clear age gradient (Supplementary Table S14): OR decreased from 1.51 (1.30–1.76) in <40 years to 0.98 (0.88–1.09) in ≥70 years, consistent with the primary analysis and the analysis excluding statin users. At the overall level, the OR for LDL-C increased modestly from 1.12 to 1.16 after handling statin use (Supplementary Table S15). The distinct age-modified patterns remained robust across all sensitivity analyses, including the three strategies for handling statin use (Supplementary Tables S14, S15) and additional tests (Supplementary Tables S4–S7, S11–S13). Further adjustment for IMTmax resulted in only a marginal attenuation of the effect estimates (Supplementary Table S7). Moreover, replacing the TG/HDL-C ratio with the TyG index yielded consistent directional results (Supplementary Tables S11–S13). Within the plaque-positive subgroup (*n* = 6,657), elevated LDL-C emerged as an independent determinant of hypoechoic plaque morphology (OR = 1.23, 95% CI: 1.16–1.30; *P* < 0.001). In contrast, the TG/HDL-C ratio showed no statistically significant association with hypoechoic plaque phenotype (OR = 1.06, 95% CI: 0.99–1.14, *P* = 0.054). This null association was confirmed when the TyG index was used as a substitute for the TG/HDL-C ratio (OR = 0.99, 95% CI: 0.89–1.09, *P* = 0.795; Supplementary Tables S9, S13).

### E-value analysis

For the positive associations between LDL-C and carotid plaque in younger age groups, E-values were computed to quantify the potential impact of unmeasured confounding (Supplementary Table S16). In the <40 age group (OR = 1.51, 95% CI: 1.30–1.76), the E-value was 2.39 (CI lower bound: 1.92), indicating that an unmeasured confounder would need to be associated with both LDL-C and plaque by a risk ratio of at least 1.92 to fully explain away the observed association. In the 40–49 age group (OR = 1.21, 95% CI: 1.11–1.33), the corresponding E-value was 1.71 (CI lower bound: 1.46). Given that the three strategies for handling statin use yielded nearly identical ORs in the younger age groups (Supplementary Table S14), the E-values calculated based on the primary analysis are representative. However, E-value analysis is a sensitivity tool and cannot replace direct adjustment for measured confounders; residual confounding from unmeasured lifestyle and clinical factors remains possible.

## Discussion

In this large-scale Chinese health check-up cohort, both LDL-C and the TG/HDL-C ratio were independently associated with carotid plaque. Crucially, formal interaction testing and continuous age comparisons revealed distinct age-modified patterns for these two lipid parameters. The association of LDL-C with carotid plaque was most pronounced in younger individuals but attenuated progressively with advancing age, becoming non-significant after 60 years in this cross-sectional setting. These findings should be interpreted as hypothesis-generating and require prospective validation. In contrast, the TG/HDL-C ratio demonstrated a relatively stable association across age strata, with the numerically highest ORs observed in the <40 and 50–59 age groups (OR = 1.20 and 1.15, respectively; *P* for interaction = 0.544). The lipid profile associated with hypoechoic plaque phenotype appeared specific to LDL-C, as neither the TG/HDL-C ratio nor the TyG index reached significance for this outcome.

The observation that the magnitude of LDL-C's association attenuates with age echoes recent findings in cardiovascular event research (31). However, it is imperative to emphasize that the cross-sectional design of this study precludes us from distinguishing true biological effect modification from survival bias. Individuals highly susceptible to LDL-C may have experienced clinical events at younger ages or initiated intensive lipid-lowering therapy, leading to their selective exclusion from our current elderly survivor cohort. Consequently, the LDL-C distribution in the older group may be skewed. Our data support this speculation: the median LDL-C level was slightly lower and the rate of suspected statin use was higher in the elderly Future longitudinal studies employing repeated LDL-C measurements are warranted to disentangle the confounding effects of aging, cohort effects, and survival bias. Moreover, the competing risks posed by non-lipid factors—such as hypertension, vascular calcification, and chronic inflammation—intensify with age (31), potentially diluting the independent contribution of LDL-C. The attenuated cross-sectional association in older age groups does not negate the causal role of LDL-C in atherogenesis. Competing risk factors, survival bias, and selective attrition from statin therapy likely diluted the association in this age group. Therefore, the age-dependent trends observed herein should be regarded as an important hypothesis requiring validation in prospective cohort studies.

LDL-C as a single marker has inherent limitations. Apolipoprotein B (ApoB), which reflects the total number of atherogenic lipoprotein particles, is considered by some investigators to be a superior predictor of cardiovascular risk compared to LDL-C alone (32). Although ApoB was not directly measured in this study, the metabolic milieu reflected by an elevated TG/HDL-C ratio—characterized by increased remnant lipoproteins, decreased HDL-C, and a predominance of small dense LDL particles—is closely associated with elevated ApoB and increased small dense LDL burden. Thus, the stable association between TG/HDL-C ratio and carotid plaque observed across age strata in this study may be partly mediated through its role as an integrated marker of small dense LDL phenotype and metabolic syndrome. Future studies incorporating ApoB and LDL particle size measurements are warranted to further elucidate these relationships. Unlike LDL-C, the TG/HDL-C ratio demonstrated a stable trajectory in relation to carotid plaque across the lifespan, with the numerically highest ORs observed in the <40 and 50–59 age groups (OR = 1.20 and 1.15, respectively). Given the non-significant age interaction (*P* = 0.544), this numerical variation should be interpreted as a descriptive pattern rather than evidence of age-specific effect modification. The TG/HDL-C ratio functions as a composite indicator of the atherogenic dyslipidemic phenotype, integrating the triad of elevated triglyceride-rich remnants, low HDL-C, and insulin resistance-mediated proliferation of small dense LDL particles (9, 10). The prevalence of such dysmetabolic state is highest among middle-aged adults, coinciding with the period of maximal vulnerability to metabolic syndrome. Curiously, the observed association was attenuated and non-significant in the 40–49 year-old cohort. Whether this decade represents a critical window for metabolic risk transition warrants further exploration in larger longitudinal studies. From a public health perspective, the TG/HDL-C ratio is practical. It can be derived directly from standard lipid profiles at no additional cost and may serve as an adjunctive marker for metabolic risk assessment in health check-up settings. For individuals with controlled LDL-C but elevated TG/HDL-C ratios, our findings suggest the need for further metabolic risk assessment. It is important to clarify that the concurrent independent associations of both lipids during mid-life do not imply synergistic or additive effects; confirming such interactions would require specific interaction analyses within age strata, which was beyond the scope of this study. Nevertheless, the observed “stage-specific pattern”—where LDL-C dominates in early life and metabolic factors gain prominence in mid-life—provides epidemiological clues supporting the “shifting drivers” hypothesis of atherosclerotic development. This stage-specific pattern—a stronger LDL-C association in early life and a numerically higher TG/HDL-C OR in mid-life—provides preliminary epidemiological clues consistent with the hypothesized “shifting drivers” pattern of atherosclerotic development.

An independent cross-sectional association with hypoechoic plaque phenotype was observed exclusively for LDL-C within the subpopulation with preexisting plaques. Conversely, the TyG index showed no significant association. This finding is consistent with the pathological hallmark of hypoechoic plaques—the lipid-rich necrotic core (33)—and suggests a potential role for LDL-C in the cross-sectional presentation of this vulnerable plaque phenotype. However, this interpretation is constrained by the lack of formal inter-observer reliability assessment for hypoechoic plaque grading and the cross-sectional design, which precludes evaluation of plaque progression or vulnerability over time. Our findings should be interpreted in light of certain constraints. The inability to establish temporality inherent to cross-sectional studies limits causal claims, and the intertwined nature of age, period, and cohort effects remains unraveled. Notably, the lower LDL-C levels and higher rates of suspected statin use observed in participants aged ≥70 years provide indirect evidence for survival bias or treatment bias. Highly susceptible individuals may have been selectively excluded from the elderly cohort due to prior clinical events or intensive lipid-lowering therapy, potentially leading to an underestimation of LDL-C's true impact in older populations. Thus, the observed age-dependent attenuation should be interpreted as a composite result of both true biological effect modification and selective survival. Second, the single-center health check-up setting limits generalizability. A selection bias towards individuals with higher education levels, better economic standing, and greater health-seeking behavior—primarily the employed and retired—was evident in our sample compared to the unselected general population. This sampling predisposition curtails the generalizability of our findings to socioeconomically marginalized populations, rural inhabitants, and the extreme elderly. Additionally, the absence of granular lifestyle data (smoking, diet, etc.) raises concerns regarding unmeasured confounding. However, such factors would typically act as positive confounders, and their omission would tend to overestimate rather than create spurious associations (34). The observed ORs were moderate (ORs ∼1.2–1.5), and E-value analysis (Supplementary Table S16) indicated that in the <40 age group, an unmeasured confounder would need a strength of association of 2.39 (CI lower bound: 1.92) with both LDL-C and plaque to fully explain away the association. Thus, while residual confounding cannot be fully excluded, it is unlikely to reverse the core qualitative finding of a stronger LDL-C association in younger age groups. Future prospective studies with serial lipid measurements and comprehensive confounder ascertainment (e.g., smoking, diet, physical activity, and medication history) are warranted to validate these observations and establish temporal relationships. Furthermore statin use was inferred rather than pharmacologically verified, which may have led to exposure misclassification. Furthermore, although standardized definitions were applied, inter-observer reliability for hypoechoic plaque assessment was not formally quantified, introducing potential measurement error. Finally, the limited discriminative ability of individual lipid parameters (AUC ≈ 0.52–0.53; Supplementary Figure S2) underscores that single biomarkers are insufficient for precise risk stratification. This highlights the necessity of developing multivariable risk prediction tools rather than relying on isolated lipid cutoffs in clinical practice.

These findings may have implications for risk assessment in health check-up settings. The relatively strong LDL-C association in the younger cohort (OR = 1.51) suggests that the contribution of early-life dyslipidemia to atherosclerosis warrants closer attention in prevention frameworks. Current guidelines already emphasize earlier lipid screening for younger populations (35); our data provide additional cross-sectional support for this approach. Whether intensified lipid management in younger adults can modify long-term plaque burden remains a question for prospective studies.

The TG/HDL-C ratio, which is derived from standard lipid panels at no additional cost, may serve as an adjunctive marker for metabolic risk assessment in mid-life health check-ups. For individuals with controlled LDL-C but elevated TG/HDL-C ratios, further metabolic evaluation may be considered. The observed stage-specific pattern—a stronger LDL-C association in early life and a numerically higher TG/HDL-C OR in mid-life—provides preliminary epidemiological clues consistent with the hypothesized “shifting drivers” pattern of atherosclerotic development. Whether these patterns translate to age-specific intervention strategies requires prospective validation.

Future longitudinal studies are needed to validate the age-modified patterns identified herein. Exploring the integration of age-interaction effects of LDL-C and the TG/HDL-C ratio into vascular biological age models (36, 37) may provide insights into whether early-life lipid profiles contribute to accelerated vascular aging.

## Conclusion

This cross-sectional investigation of 21,227 Chinese adults recruited from a health check-up center characterized the effect modification of age on the lipid-plaque association through formal interaction tests. Two distinct age-dependent patterns emerged. The association of LDL-C with carotid plaque was most pronounced in younger individuals but attenuated progressively with advancing age, becoming non-significant in older groups. In contrast, the TG/HDL-C association remained stable across age strata. Additionally, LDL-C was associated with hypoechoic plaque phenotype, a cross-sectional surrogate of vulnerability. A key constraint of this design is the inability to distinguish true effect modification from survival bias and cohort effects. Prospective studies with longitudinal lipid measurements are warranted to validate these hypotheses. Within these limitations, our findings provide epidemiological clues supporting the hypothesis that age-specific lipid assessment strategies may merit further investigation. The TG/HDL-C ratio, derived from standard lipid panels at no additional cost, may serve as a practical adjunctive marker for metabolic cardiovascular risk assessment in health check-up settings.

## Data Availability

The raw data supporting the conclusions of this article will be made available by the authors, without undue reservation.
