# Supplementary material for: Differential age-related patterns in the associations of LDL-C, TG/HDL-C ratio, and carotid plaque: a large-scale cross-sectional study in a Chinese health check-up population
Source: Front Cardiovasc Med. 2026 Jul 20;13:1887660. doi: 10.3389/fcvm.2026.1887660 (PMC13429426; doi:10.3389/fcvm.2026.1887660)
Supplement: Supplementary file 1 [file Datasheet1.docx]

**Table S1. Baseline characteristics by carotid atherosclerosis severity**

| **Characteristic** | **Normal**  N = 11,895^1^ | **IMT thickening only**  N = 2,675^1^ | **Plaque present**  N = 6,657^1^ | **p-value**^2^ |
| --- | --- | --- | --- | --- |
| Age (years) | 42 [35, 51] | 59 [52, 67] | 60 [52, 70] | <0.001 |
| Gender |  |  |  | <0.001 |
| Female | 4,779 (40%) | 974 (36%) | 2,154 (32%) |  |
| Male | 7,116 (60%) | 1,701 (64%) | 4,503 (68%) |  |
| Height (cm) | 166 [160, 172] | 165 [158, 170] | 165 [158, 170] | <0.001 |
| Weight (kg) | 66 [57, 75] | 67 [59, 75] | 65 [58, 73] | <0.001 |
| BMI (kg/m²) | 24.0 [21.9, 26.1] | 24.8 [22.8, 27.1] | 24.3 [22.3, 26.2] | <0.001 |
| SBP (mmHg) | 121 [111, 132] | 132 [121, 143] | 132 [121, 144] | <0.001 |
| DBP (mmHg) | 74 [66, 82] | 78 [70, 85] | 77 [70, 85] | <0.001 |
| Glucose (mmol/L) | 5.16 [4.88, 5.52] | 5.43 [5.06, 5.99] | 5.46 [5.08, 6.05] | <0.001 |
| Total cholesterol (mmol/L) | 4.96 [4.40, 5.60] | 5.21 [4.56, 5.86] | 5.17 [4.47, 5.85] | <0.001 |
| Triglycerides (mmol/L) | 1.30 [0.90, 1.92] | 1.42 [1.02, 1.97] | 1.40 [1.02, 1.99] | <0.001 |
| HDL-C (mmol/L) | 1.27 [1.08, 1.50] | 1.25 [1.07, 1.48] | 1.25 [1.07, 1.46] | <0.001 |
| LDL-C (mmol/L) | 3.03 [2.50, 3.57] | 3.25 [2.63, 3.81] | 3.15 [2.52, 3.76] | <0.001 |
| Non-HDL-C (mmol/L) | 3.65 [3.09, 4.27] | 3.90 [3.27, 4.56] | 3.86 [3.19, 4.53] | <0.001 |
| TG/HDL-C ratio | 1.03 [0.63, 1.70] | 1.12 [0.72, 1.75] | 1.13 [0.72, 1.74] | <0.001 |
| LDL-C/HDL-C ratio | 2.41 [1.83, 3.00] | 2.58 [1.98, 3.20] | 2.50 [1.89, 3.13] | <0.001 |
| IMTmax (mm) | 0.70 [0.70, 0.80] | 1.10 [1.00, 1.20] | 1.00 [0.90, 1.10] | <0.001 |
| Suspected statin use | 157 (1.3%) | 111 (4.1%) | 416 (6.2%) | <0.001 |

*Baseline characteristics by carotid atherosclerosis severity. Data are median [IQR] or n (%). Normal: IMT<1.0mm, no plaque; IMT thickening: IMT≥1.0mm, no plaque. P values: Kruskal-Wallis or χ² test. Abbreviations: BMI, body mass index; DBP, diastolic blood pressure; HDL-C, high-density lipoprotein cholesterol; IMT, intima-media thickness; IMTmax, maximum IMT; LDL-C, low-density lipoprotein cholesterol; Non-HDL-C, non-HDL cholesterol; SBP, systolic blood pressure; TG, triglycerides.*

**Table S2. Univariate logistic regression for carotid plaque**

| Characteristic | Crude OR (95% CI) | p-value |
| --- | --- | --- |
| Age (per 1 year) | 1.09 (1.09-1.09) | <0.001 |
| Male sex (vs. Female) | 1.36 (1.28-1.45) | <0.001 |
| BMI (per 1 kg/m2) | 1.00 (0.99-1.01) | 0.598 |
| SBP (per 1 mmHg) | 1.03 (1.03-1.03) | <0.001 |
| Glucose (per 1 mmol/L) | 1.33 (1.29-1.36) | <0.001 |
| LDL-C (per 1 mmol/L) | 1.09 (1.05-1.12) | <0.001 |
| TG/HDL-C ratio (per 1 unit) | 1.07 (1.04-1.1) | <0.001 |

*Univariate logistic regression for carotid plaque. Crude OR (95% CI). Abbreviations: BMI, body mass index; LDL-C, low-density lipoprotein cholesterol; SBP, systolic blood pressure; TG/HDL-C, triglyceride to HDL-C ratio.*

**Table S3. ROC analysis for LDL-C and TG/HDL-C ratio in discriminating carotid plaque**

| Marker | AUC | AUC（95%CI） | Optimal_cutoff | Sensitivity | Specificity |
| --- | --- | --- | --- | --- | --- |
| LDL-C | 0.521 | 0.513-0.530 | 3.44 | 0.376 | 0.674 |
| TG/HDL-C ratio | 0.532 | 0.524-0.540 | 0.75 | 0.739 | 0.325 |

*ROC analysis for LDL-C and TG/HDL-C ratio. AUC: area under curve. Optimal cutoff by Youden index. Abbreviations: AUC, area under the curve; LDL-C, low-density lipoprotein cholesterol; TG/HDL-C, triglyceride to HDL-C ratio.*

**Table S4. Sensitivity analysis excluding suspected statin users (N = 20,543)**

| **Characteristic** | **OR (95% CI)**^1^ | **p-value** |
| --- | --- | --- |
| Age (per 1 year) | 1.09*** | <0.001 |
| Male sex (vs. Female) |  |  |
| Female | — |  |
| Male | 1.66*** | <0.001 |
| BMI (per 1 kg/m²) | 0.97*** | <0.001 |
| SBP (per 1 mmHg) | 1.01*** | <0.001 |
| Glucose (per 1 mmol/L) | 1.04** | 0.005 |
| LDL-C (per 1 mmol/L) | 1.16*** | <0.001 |
| TG/HDL-C ratio (per 1 unit) | 1.13*** | <0.001 |

Sensitivity analysis excluding suspected statin users (N=20,543). Models adjusted for age, sex, BMI, SBP, glucose. *p<0.05; **p<0.01; ***p<0.001. Abbreviations: BMI, body mass index; CI, confidence interval; LDL-C, low-density lipoprotein cholesterol; OR, odds ratio; SBP, systolic blood pressure; TG/HDL-C, triglyceride to HDL-C ratio.

**Table S5. Sensitivity analysis using Non-HDL-C instead of LDL-C (N = 21,227)**

| **Characteristic** | **OR (95% CI)**^1^ | **p-value** |
| --- | --- | --- |
| Age (per 1 year) | 1.09*** | <0.001 |
| Male sex (vs. Female) |  |  |
| Female | — |  |
| Male | 1.72*** | <0.001 |
| BMI (per 1 kg/m²) | 0.98*** | <0.001 |
| SBP (per 1 mmHg) | 1.01*** | <0.001 |
| Glucose (per 1 mmol/L) | 1.04** | 0.002 |
| Non-HDL-C (per 1 mmol/L) | 1.17*** | <0.001 |
| TG/HDL-C ratio (per 1 unit) | 1.06* | 0.011 |

*Sensitivity analysis using Non-HDL-C instead of LDL-C (N=21,227). Models adjusted for age, sex, BMI, SBP, glucose. Non-HDL-C = total cholesterol - HDL-C. Abbreviations: BMI, body mass index; CI, confidence interval; Non-HDL-C, non-HDL cholesterol; OR, odds ratio; SBP, systolic blood pressure; TG/HDL-C, triglyceride to HDL-C ratio.*

**Table S6. Sensitivity analysis excluding participants with glucose ≥ 7.0 mmol/L (N = 19,700)**

| **Characteristic** | **OR (95% CI)**^1^ | **p-value** |
| --- | --- | --- |
| Age (per 1 year) | 1.09*** | <0.001 |
| Male sex (vs. Female) |  |  |
| Female | — | — |
| Male | 1.68*** | <0.001 |
| BMI (per 1 kg/m²) | 0.97*** | <0.001 |
| SBP (per 1 mmHg) | 1.01*** | <0.001 |
| Glucose (per 1 mmol/L) | 1.04 | 0.3 |
| LDL-C (per 1 mmol/L) | 1.13*** | <0.001 |
| TG/HDL-C ratio (per 1 unit) | 1.13*** | <0.001 |

*Table S6. Sensitivity analysis excluding participants with glucose ≥7.0 mmol/L (N=19,700). Models adjusted for age, sex, BMI, SBP, glucose. Abbreviations: BMI, body mass index; CI, confidence interval; LDL-C, low-density lipoprotein cholesterol; OR, odds ratio; SBP, systolic blood pressure; TG/HDL-C, triglyceride to HDL-C ratio.*

**Table S7. Sensitivity analysis additionally adjusting for IMTmax (N = 21,227)**

| **Characteristic** | **OR (95% CI)**^1^ | **p-value** |
| --- | --- | --- |
| Age (per 1 year) | 1.06*** | <0.001 |
| Male sex (vs. Female) |  |  |
| Female | — | — |
| Male | 1.55*** | <0.001 |
| BMI (per 1 kg/m²) | 0.96*** | <0.001 |
| SBP (per 1 mmHg) | 1.00*** | <0.001 |
| Glucose (per 1 mmol/L) | 1.03* | 0.023 |
| LDL-C (per 1 mmol/L) | 1.08*** | <0.001 |
| TG/HDL-C ratio (per 1 unit) | 1.11*** | <0.001 |
| IMTmax (per 1 mm) | 19.0*** | <0.001 |

*Sensitivity analysis additionally adjusting for IMTmax (N=21,227). Models adjusted for age, sex, BMI, SBP, glucose, LDL-C, TG/HDL-C, and IMTmax. Abbreviations: BMI, body mass index; CI, confidence interval; IMTmax, maximum intima-media thickness; LDL-C, low-density lipoprotein cholesterol; OR, odds ratio; SBP, systolic blood pressure; TG/HDL-C, triglyceride to HDL-C ratio.*

**Table S8. Multivariable logistic regression for IMT thickening (IMT ≥ 1.0 mm)**

| **Characteristic** | **OR (95% CI)**^1^ | **p-value** |
| --- | --- | --- |
| Age (per 1 year) | 1.13*** | <0.001 |
| Male sex (vs. Female) |  |  |
| Female | — | — |
| Male | 1.43*** | <0.001 |
| BMI (per 1 kg/m²) | 1.07*** | <0.001 |
| SBP (per 1 mmHg) | 1.01*** | <0.001 |
| Glucose (per 1 mmol/L) | 1.03* | 0.020 |
| LDL-C (per 1 mmol/L) | 1.21*** | <0.001 |
| TG/HDL-C ratio (per 1 unit) | 1.04 | 0.10 |

*Multivariable logistic regression for IMT thickening (IMT ≥1.0 mm, no plaque). Models adjusted for age, sex, BMI, SBP, glucose. Abbreviations: BMI, body mass index; CI, confidence interval; IMT, intima-media thickness; LDL-C, low-density lipoprotein cholesterol; OR, odds ratio; SBP, systolic blood pressure; TG/HDL-C, triglyceride to HDL-C ratio.*

**Table S9. Multivariable analysis for hypoechoic plaque among participants with carotid plaque (N = 6,657)**

| **Characteristic** | **OR (95% CI)**^1^ | **p-value** |
| --- | --- | --- |
| Age (per 1 year) | 0.98*** | <0.001 |
| Male sex (vs. Female) |  |  |
| Female | — | — |
| Male | 1.67*** | <0.001 |
| BMI (per 1 kg/m²) | 0.98* | 0.016 |
| SBP (per 1 mmHg) | 1.00 | 0.2 |
| Glucose (per 1 mmol/L) | 0.98 | 0.3 |
| LDL-C (per 1 mmol/L) | 1.23*** | <0.001 |
| TG/HDL-C ratio (per 1 unit) | 1.06 | 0.054 |

*Multivariable analysis for hypoechoic plaque among participants with carotid plaque (N=6,657). Hypoechoic plaque defined as hypoechoic area ≥50% of total plaque area. Models adjusted for age, sex, BMI, SBP, glucose. Abbreviations: BMI, body mass index; CI, confidence interval; LDL-C, low-density lipoprotein cholesterol; OR, odds ratio; SBP, systolic blood pressure; TG/HDL-C, triglyceride to HDL-C ratio.*

**Table S10. Full age-stratified multivariable associations for all covariates with carotid plaque**

| Characteristic | <40 | 40-49 | 50-59 | 60-69 | ≥70 |
| --- | --- | --- | --- | --- | --- |
|  | N=5324 | N=5033 | N=5317 | N=3151 | N=2402 |
| Age (per 1 year) | 1.18 (1.14–1.23) P < 0.001 | 1.12 (1.09–1.15) P < 0.001 | 1.08 (1.06–1.1) P < 0.001 | 1.05 (1.03–1.08) P < 0.001 | 1.02 (1.01–1.04) P = 0.005 |
| Male sex | 1.76 (1.24–2.52) P = 0.002 | 1.84 (1.53–2.22) P < 0.001 | 1.7 (1.5–1.93) P < 0.001 | 1.91 (1.64–2.23) P < 0.001 | 1.25 (1.04–1.49) P = 0.016 |
| BMI (kg/m²) | 0.96 (0.91–1) P = 0.052 | 0.96 (0.93–0.99) P = 0.003 | 0.96 (0.94–0.98) P < 0.001 | 0.96 (0.94–0.99) P = 0.007 | 1.01 (0.98–1.04) P = 0.477 |
| SBP (mmHg) | 1 (0.99–1.01) P = 0.723 | 1.02 (1.01–1.02) P < 0.001 | 1 (1–1.01) P = 0.253 | 1.01 (1–1.01) P = 0.002 | 1.01 (1–1.01) P = 0.017 |
| Glucose (mmol/L) | 1.01 (0.85–1.17) P = 0.887 | 1.05 (0.99–1.12) P = 0.084 | 1.05 (1.01–1.1) P = 0.019 | 1.03 (0.98–1.08) P = 0.29 | 1.01 (0.96–1.08) P = 0.65 |
| LDL-C (mmol/L) | 1.51 (1.3–1.76) P < 0.001 | 1.21 (1.11–1.33) P < 0.001 | 1.1 (1.03–1.18) P = 0.003 | 1.01 (0.94–1.1) P = 0.749 | 0.94 (0.85–1.03) P = 0.162 |
| TG/HDL-C ratio | 1.2 (1.05–1.37) P = 0.008 | 1.08 (1–1.17) P = 0.051 | 1.15 (1.08–1.23) P < 0.001 | 1.02 (0.92–1.12) P = 0.741 | 0.99 (0.88–1.12) P = 0.874 |

*Full age-stratified multivariable associations for all covariates with carotid plaque. Values are OR (95% CI). Models within each stratum adjusted for continuous age, sex, BMI, SBP, glucose (except when stratified by the variable itself). Abbreviations: BMI, body mass index; LDL-C, low-density lipoprotein cholesterol; OR, odds ratio; SBP, systolic blood pressure; TG/HDL-C, triglyceride to HDL-C ratio.*

**Table S11. Multivariable logistic regression for carotid plaque using TyG index instead of TG/HDL-C ratio (N=21,227)**

| Characteristic | OR (95% CI) | p-value |
| --- | --- | --- |
| Age (per 1 year) | 1.09 (1.09–1.09)*** | <0.001 |
| Female | — | — |
| Male | 1.70 (1.57–1.83)*** | <0.001 |
| BMI (per 1 kg/m²) | 0.97 (0.96–0.99)*** | <0.001 |
| SBP (per 1 mmHg) | 1.01 (1–1.01)*** | <0.001 |
| LDL-C (per 1 mmol/L) | 1.11 (1.06–1.15)*** | <0.001 |
| TyG index (per 1 unit) | 1.25 (1.17–1.33)*** | <0.001 |

*Multivariable logistic regression for carotid plaque using TyG index instead of TG/HDL-C ratio (N=21,227). TyG = ln[TG(mg/dL)×glucose(mg/dL)/2]. Models adjusted for age, sex, BMI, SBP, LDL-C. Fasting glucose not adjusted due to collinearity. Abbreviations: BMI, body mass index; CI, confidence interval; LDL-C, low-density lipoprotein cholesterol; OR, odds ratio; SBP, systolic blood pressure; TyG, triglyceride-glucose index.*

**Table S12. Age-stratified multivariable association of TyG index with carotid plaque**

| Age Group | N | Events | OR (95% CI) | p-value |
| --- | --- | --- | --- | --- |
| <40 | 5,324 | 284 | 1.29 (0.99-1.68) | 0.0571 |
| 40-49 | 5,033 | 935 | 1.22 (1.05-1.41) | 0.0097 |
| 50-59 | 5,317 | 1,980 | 1.28 (1.15-1.44) | <0.0001 |
| 60-69 | 3,151 | 1,782 | 1.13 (0.98-1.31) | 0.1015 |
| ≥70 | 2,402 | 1,676 | 1.03 (0.87-1.23) | 0.7166 |

*Age-stratified association of TyG index with carotid plaque. Models within each stratum adjusted for age, sex, BMI, SBP, LDL-C. Fasting glucose not adjusted. Abbreviations: CI, confidence interval; LDL-C, low-density lipoprotein cholesterol; OR, odds ratio; SBP, systolic blood pressure; TyG, triglyceride-glucose index.*

**Table S13. Multivariable analysis for hypoechoic plaque using TyG index (among participants with carotid plaque)(N = 6,657)**

| Characteristic | OR (95% CI) | p-value |
| --- | --- | --- |
| Age (per 1 year) | 0.98 (0.98–0.98)*** | <0.001 |
| Female | — | — |
| Male | 1.69 (1.50–1.90)*** | <0.001 |
| BMI (per 1 kg/m²) | 0.98 (0.96–1.00) | 0.0602 |
| SBP (per 1 mmHg) | 1.00 (1.00–1.01) | 0.1782 |
| LDL-C (per 1 mmol/L) | 1.23 (1.16–1.30)*** | <0.001 |
| TyG index (per 1 unit) | 0.99 (0.89-1.09) | 0.7954 |

*Multivariable analysis for hypoechoic plaque using TyG index among participants with carotid plaque (N=6,657). Outcome: hypoechoic plaque (≥50% hypoechoic area). Models adjusted for age, sex, BMI, SBP, LDL-C. Fasting glucose not adjusted. Abbreviations: BMI, body mass index; CI, confidence interval; LDL-C, low-density lipoprotein cholesterol; OR, odds ratio; SBP, systolic blood pressure; TyG, triglyceride-glucose index.*

**Table S14. Age-stratified associations of LDL-C and TG/HDL-C ratio with carotid plaque across three strategies for handling statin use**

|  |  |  | LDL-C OR (95% CI) | | | TG/HDL-C Ratio OR (95% CI) | | |
| --- | --- | --- | --- | --- | --- | --- | --- | --- |
| Age group | N | Plaque events | Main (unadj.) | + Adj. for statin | Excl. statin users | Main (unadj.) | + Adj. for statin | Excl. statin users |
| <40 | 5,324 | 284 | 1.51 (1.30-1.76)*** | 1.51 (1.30-1.76)*** | 1.51 (1.30-1.76)*** | 1.20 (1.05-1.37)** | 1.20 (1.05-1.37)** | 1.20 (1.05-1.37)** |
| 40-49 | 5,033 | 935 | 1.21 (1.11-1.33)*** | 1.21 (1.11-1.33)*** | 1.21 (1.11-1.33)*** | 1.08 (1.00-1.17) | 1.08 (1.00-1.18) | 1.08 (1.00-1.17) |
| 50-59 | 5,317 | 1,980 | 1.10 (1.03-1.18)** | 1.13 (1.05-1.21)*** | 1.10 (1.03-1.18)** | 1.15 (1.08-1.23)*** | 1.15 (1.08-1.23)*** | 1.15 (1.08-1.23)*** |
| 60-69 | 3,151 | 1,782 | 1.01 (0.94-1.10) | 1.03 (0.94-1.12) | 1.01 (0.94-1.10) | 1.02 (0.92-1.12) | 1.02 (0.92-1.12) | 1.02 (0.92-1.12) |
| ≥70 | 2,402 | 1,676 | 0.94 (0.85-1.03) | 0.98 (0.88-1.09) | 0.94 (0.85-1.03) | 0.99 (0.88-1.12) | 0.99 (0.88-1.12) | 0.99 (0.88-1.12) |

*Abbreviations: CI, confidence interval; LDL-C, low-density lipoprotein cholesterol; OR, odds ratio; TG/HDL-C, triglyceride to high-density lipoprotein cholesterol ratio.*

*All models were adjusted for age (continuous within each stratum), sex, BMI, SBP, fasting glucose, and the other lipid variable. Suspected statin use was defined as meeting either of the following criteria: (1) self-reported use of lipid-lowering medication, or (2) LDL-C < 1.8 mmol/L in the absence of other known etiologies for low LDL-C (e.g., cirrhosis, malignancy, severe malnutrition) based on medical history. "Main (unadj.)" refers to the primary analysis without adjustment for statin use; "+ Adj. for statin" refers to the full population with additional adjustment for suspected statin use; "Excl. statin users" refers to the sensitivity analysis excluding suspected statin users (n = 20,543). In the <40 age group, results were identical across the three strategies because no participants met the criteria for suspected statin use.*

**p < 0.05; ** p < 0.01; *** p < 0.001.*

**Table S15. Sensitivity analysis of lipid associations with carotid plaque across three strategies for handling statin use (overall population)**

| Strategy | LDL-C OR (95% CI) | TG/HDL-C Ratio OR (95% CI) |
| --- | --- | --- |
| Main (unadjusted) | 1.12 (1.08-1.17) | 1.11 (1.07-1.15) |
| Main + adjusted for statin | 1.16 (1.11-1.21) | 1.11 (1.07-1.15) |
| Excluding statin users | 1.16 (1.09-1.23) | 1.13 (1.07-1.19) |

*Abbreviations: CI, confidence interval; LDL-C, low-density lipoprotein cholesterol; OR, odds ratio; TG/HDL-C, triglyceride to high-density lipoprotein cholesterol ratio.*

*All models were adjusted for age, sex, BMI, SBP, fasting glucose, and the other lipid variable. Suspected statin use was defined as meeting either of the following criteria: (1) self-reported use of lipid-lowering medication, or (2) LDL-C < 1.8 mmol/L in the absence of other known etiologies for low LDL-C (e.g., cirrhosis, malignancy, severe malnutrition) based on medical history. "Main (unadjusted)" refers to the primary analysis without adjustment for statin use; "Main + adjusted for statin" refers to the full population with additional adjustment for suspected statin use; "Excluding statin users" refers to the sensitivity analysis excluding suspected statin users (n = 20,543).*

**Table S16. E-value analysis for unmeasured confounding for the association between LDL-C and carotid plaque in younger age groups**

| Age group | OR (point estimate) | 95% CI | E-value (point estimate) | E-value (CI lower bound) | Interpretation |
| --- | --- | --- | --- | --- | --- |
| <40 years | 1.51 | 1.30-1.76 | 2.39 | 1.92 | Unmeasured confounder with RR > 2.0 needed to nullify |
| 40-49 years | 1.21 | 1.11-1.33 | 1.71 | 1.46 | Unmeasured confounder with RR 1.5-2.0 needed to nullify |

*Abbreviations: CI, confidence interval; LDL-C, low-density lipoprotein cholesterol; OR, odds ratio; RR, risk ratio.*

*The E-value represents the minimum strength of association, on the risk ratio scale, that an unmeasured confounder would need to have with both the exposure and the outcome to fully explain away the observed association, conditional on the measured covariates. The E-value for the CI lower bound corresponds to the confidence interval limit closest to the null. E-values were calculated using the method of VanderWeele and Ding (2017). For the <40 age group (plaque prevalence 5.3%), the rare outcome assumption was applied. For the 40-49 age group (plaque prevalence 18.6%), E-values calculated with the rare outcome approximation were similar to those obtained when this assumption was relaxed.*

**Figure S1. Flowchart of participant selection**

*
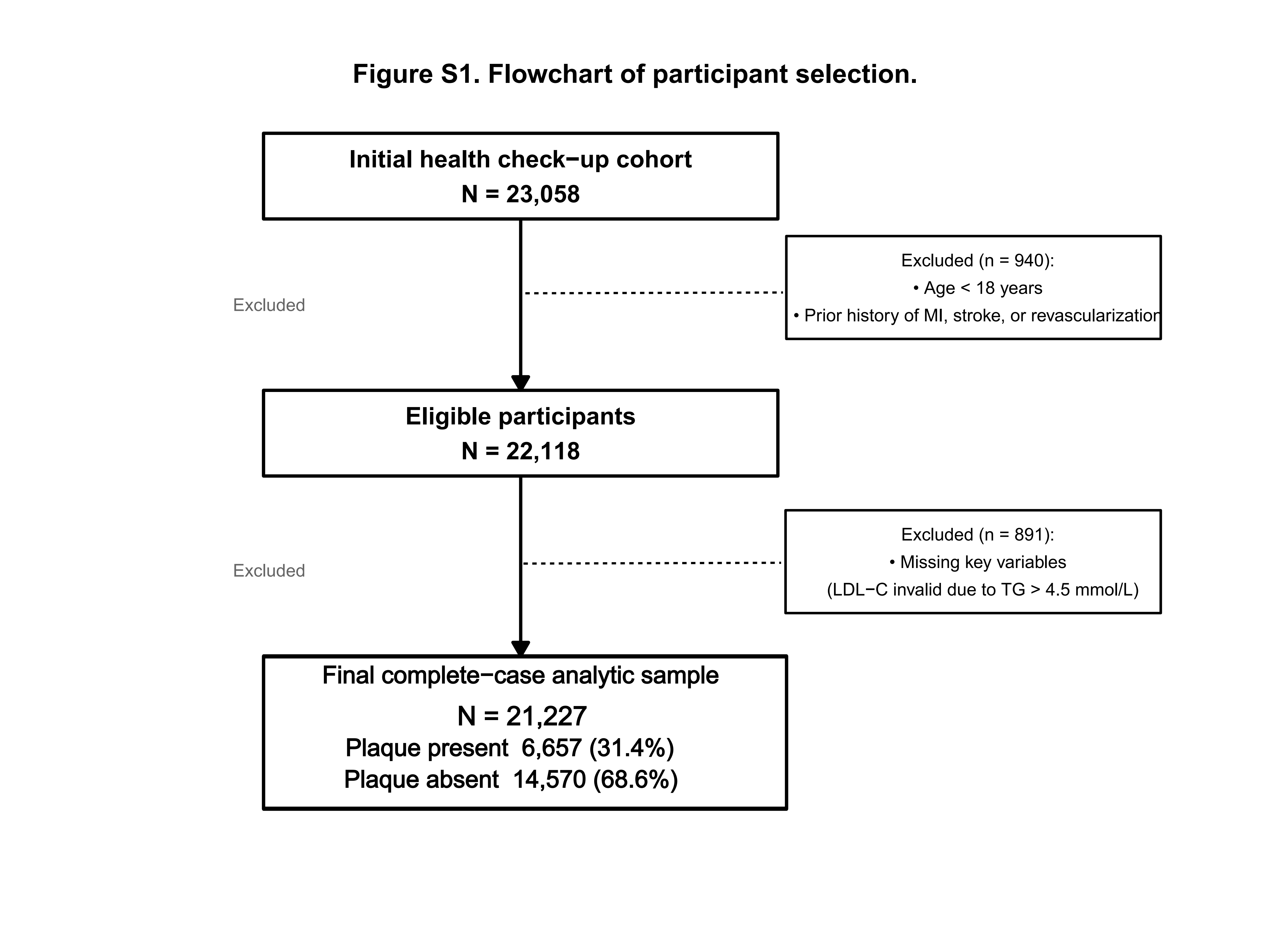
*

*Flowchart of participant selection. Initial screening: 23,058 adults. Exclusions: age <18 (none), history of cardiovascular events (not shown), missing key variables (891 with missing LDL-C). Final analysis: 21,227 participants.*

**Figure S2.ROC Curves for Discriminating Carotid Plaque**
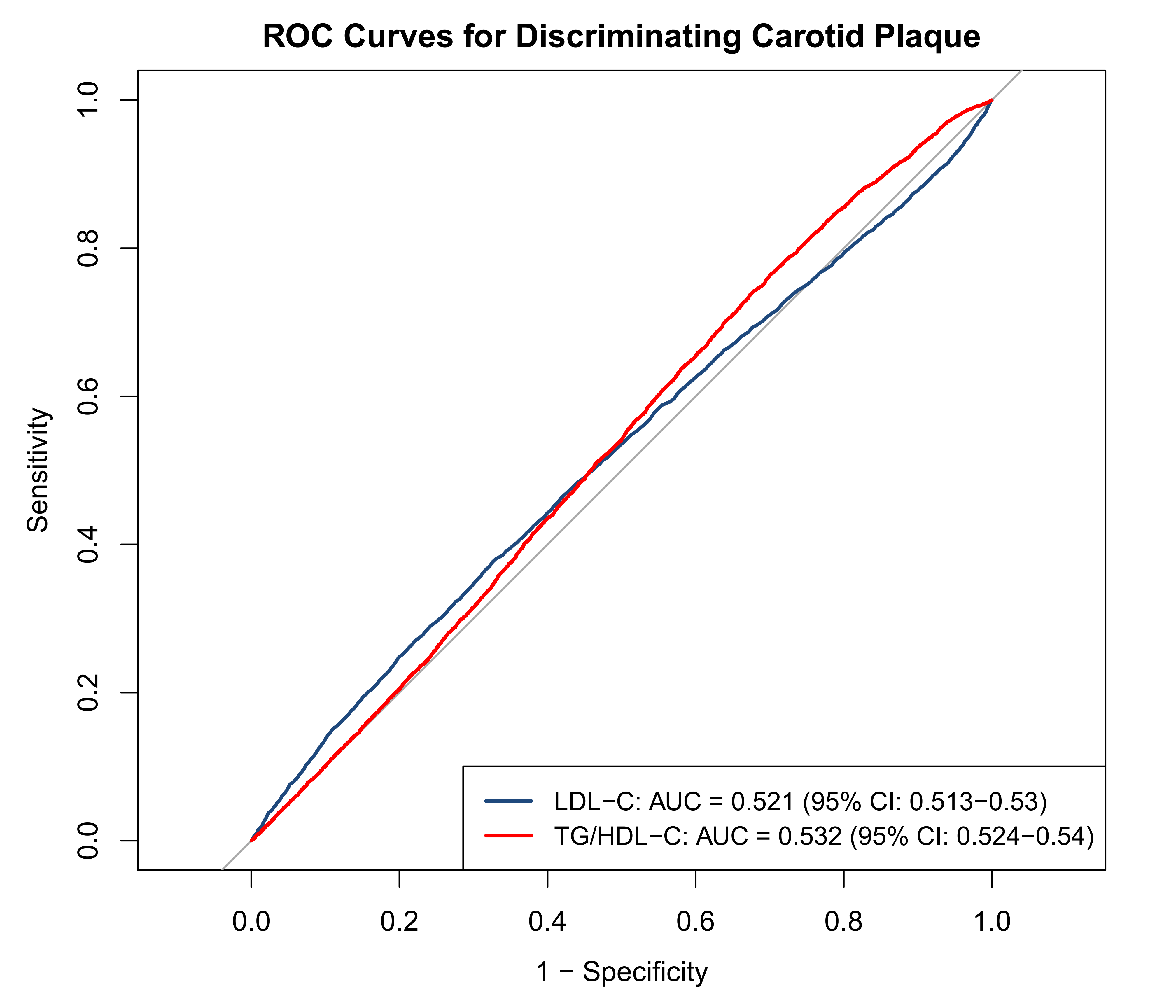


*ROC curves for discriminating carotid plaque using LDL‑C and TG/HDL‑C ratio. Diagonal dashed line: AUC=0.5. AUC values in Table S3. Abbreviations: AUC, area under curve; LDL‑C, low‑density lipoprotein cholesterol; ROC, receiver operating characteristic; TG/HDL‑C, triglyceride to HDL‑C ratio.*
